# Transcriptomic analysis reveals recovery strategies in strawberry roots after using a soil amendment in continuous cropping soil

**DOI:** 10.1186/s12870-019-2216-x

**Published:** 2020-01-03

**Authors:** Peng Chen, Yu-zhu Wang, Qi-zhi Liu, Wei-hua Li, He-qin Li, Xing-yue Li, Yun-tao Zhang

**Affiliations:** 1 Laboratory of Entomology and Nematology, College of Plant Protection, China Agricultural University, Yuan Ming-yuan West Road #2, Beijing, 100193 China; 2 Beijing Academy of Forestry and Pomology Sciences, Beijing Academy of Agriculture and Forestry Science, Beijing, 100097 China; 3 Key Laboratory of Drinking Water Science and Technology, Research Centre for Eco-Environmental Sciences, Chinese Academy of Sciences, Beijing, 100085 China; 4 Shandong Provincial Key Laboratory of Dryland Technology, College of Agronomy, Qingdao Agricultural University, Qingdao, 266109 China; 5 Institute of Plant Protection, Sichuan Academy of Agricultural Science, Chengdu, 610066 China

**Keywords:** Continuous cropping, Soil amendment, Hsp-family genes, Apoptosis, Hypersensitive response

## Abstract

**Background:**

In strawberry cultivation, continuous cropping (CC) obstacles seriously threaten production. A patented soil amendment (SA) can effectively relieve the CC obstacles to strawberry cultivation, but knowledge of the recovery mechanisms underlying this phenomenon is limited.

**Results:**

In this study, transcriptomic profiling of strawberry roots in soil with and without the SA was conducted using RNA-Seq technology to reveal gene expression changes in response to SA treatment. In total, 188 differentially expressed genes (DEGs), including 144 upregulated and 44 downregulated DEGs, were identified. SA treatment resulted in genotype-dependent responses, and the response pattern, including an overall increase in the expression of nutrient transport genes and a decrease in the expression of defense response genes, may be a possible mechanism underlying recovery strategies in strawberry roots after the application of the SA to CC soil. We also found that 9 *Hsp* genes involved in plant defense pathways were all downregulated in the SA-treated roots.

**Conclusions:**

This research indicated that strawberry plants reallocated defense resources to development when SA treatment alleviated the stress caused by a CC soil environment. The present study provides an opportunity to reveal the fundamental mechanisms of the tradeoff between growth and defense in strawberry.

## Background

Strawberry (*Fragaria* × *ananassa* Duch.), a major economic fruit, is widely cultivated in many countries [1]. Cultivated strawberry is a typical annual plant and is grown mainly under greenhouse conditions. However, the strawberry is terribly threatened by the continuous cropping (CC) problem [2–6]. Substantial agricultural losses due to CC obstacles are observed every year, and sustainable strawberry cultivation has been impeded worldwide [3].

Under CC conditions, replanting obstacles are caused by complex factors and lead to weak root systems, low productivity, and short economic lifespan [2–5, 7]. For continuously cropped soil, long-term monoculture significantly disturbs soil environment, such as reductions in land fertility, accumulations of autotoxic substances and build-up of specific microbial communities and nematode communities [2–5]. At present, soil disinfection is a common measure to control strawberry CC obstacles [8, 9]. However, the methyl bromide soil fumigants used for many years have been banned worldwide, and the existing alternative products or technologies have some limitations, such as their high toxicity, high cost, and slow decomposition [10–12]. Therefore, long-term studies are required to develop nonchemical alternatives, which will require effective integration with agricultural management [13]. A patented soil amendment (SA) product independently developed by our laboratory can relieve CC obstacles in strawberry, with results such as significantly increased strawberry plant biomass, improved soil enzyme activities and increased soil mineral nitrogen content [14]. The application of SA during strawberry engraftment can increase the fruit yield and quality of CC-produced strawberries [15].

Plants are constantly faced with variable environments and have evolved many adaptive responses to adjust to various growth conditions [16]. Yang et al. found some differentially expressed miRNAs involved in the development of the tuberous root after a *R. glutinosa* CC obstacle [17]. In our previous study, we found that several WRKY group III members might play important roles in the response to CC obstacles in strawberry [18]. However, no report is available regarding the recovery strategies in plant roots after application of the SA to CC soil. Previous studies have focused only on identifying the causative factors underlying development, yield and quality after the application of the SA [14, 15].

Although understanding the molecular mechanisms of CC tolerance in plants is necessary, it is also important to understand the recovery strategies of plants after SA application. In this study, we used RNA-Seq technology to analyze the transcriptional differences in strawberry after the application of SA to CC soil. The major objective of this work was to understand the molecular mechanisms underlying the recovery strategies of plant roots after soil improvement and to provide valuable clues for identifying genes associated with CC tolerance.

## Results

### Effects of SA on growth

**Fig. 1:**
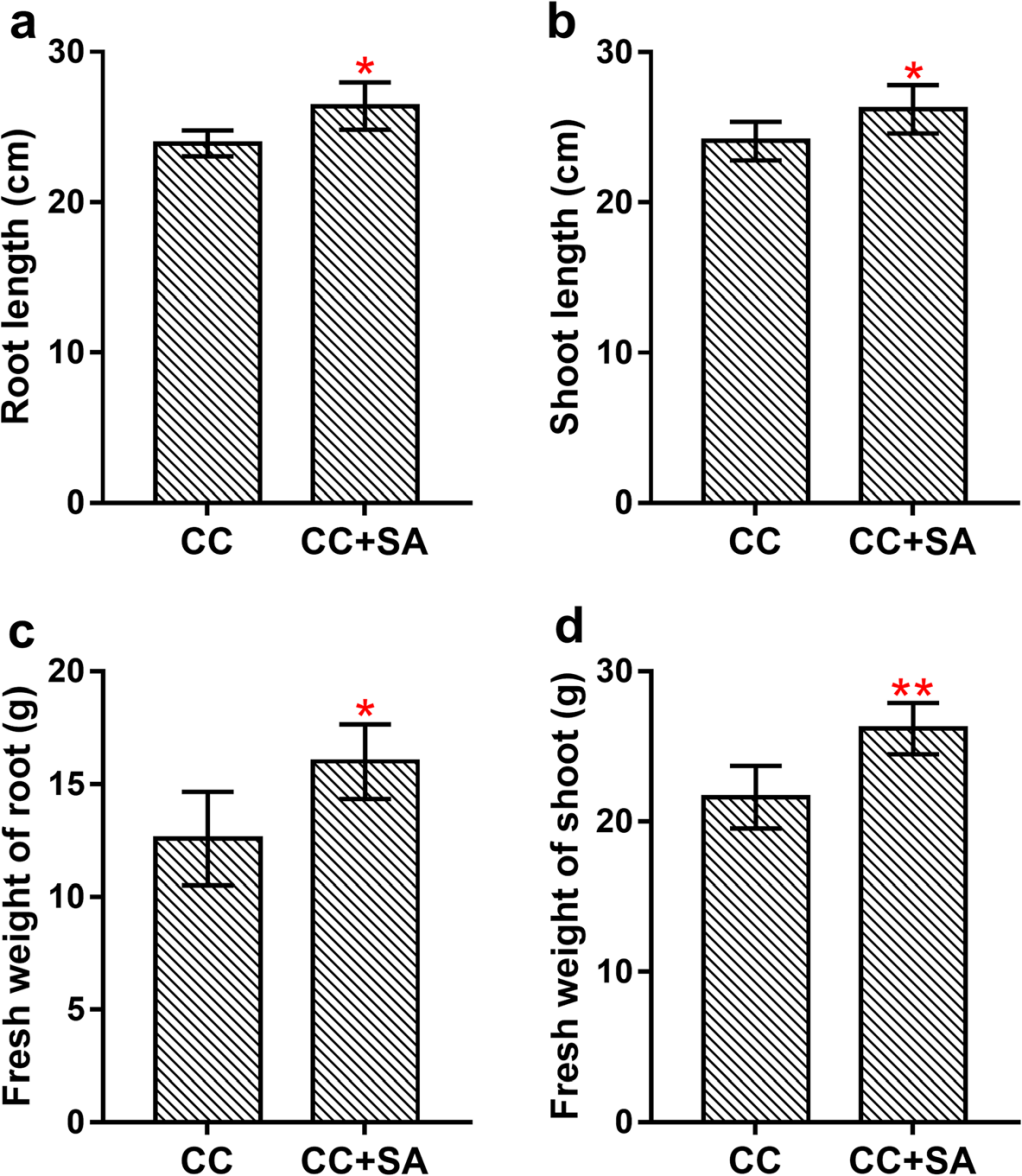
Effect of the SA on the growth of CC strawberry plants. (**a**-**b**) The length of the roots and shoots. (**c**-**d**) The fresh weight of the roots and shoots. * indicates *P* < 0.05; ** indicates *P* < 0.01

The lengths and fresh weights of the roots and shoots of strawberry plants were influenced by the SA (Fig. 1). The lengths of the roots and shoots were significantly (*P* < 0.05) stimulated by the SA, with 10.37 and 8.80% increases in comparison with those of the control, respectively (Fig. 1a-b). Furthermore, root fresh weight was significantly (*P* < 0.05) increased by 27.17%, and shoot fresh weight was extremely significantly (*P* < 0.01) stimulated by 21.17%, in the soil with the SA (Fig. 1c-d).

### Identification of differentially expressed genes (DEGs)

To obtain a general overview of differences in gene expression in strawberry roots, six libraries (CC-1, CC-2, CC-3, CC + SA-1, CC + SA-2 and CC + SA-3) were constructed for RNA-Seq. Each library produced over 25,000,000 clean bases with a Q30 percentage greater than 91.5% (Table 1). More than 72% of the clean reads mapped either to a unique location or to multiple genomic locations.

**Table 1:** Summary of the read mapping for RNA-seq

	Raw reads	Base Number	Clean Reads	GC Content (%)	Q30 (%)	Mapped Reads	Mapped Ratio (%)
CC1	7,770,204,780	7,675,408,282	25,742,493	46.49%	92.34%	19,008,544	73.84%
CC2	8,380,218,927	8,284,684,432	27,771,632	46.69%	92.22%	20,451,929	73.64%
CC3	9,018,718,832	8,931,237,260	29,900,828	47.41%	91.54%	22,380,673	74.85%
CC + SA1	7,894,932,519	7,790,719,410	26,105,035	46.13%	91.90%	18,824,278	72.11%
CC + SA2	7,618,396,035	7,551,354,150	25,279,097	46.81%	92.50%	18,946,368	74.95%
CC + SA3	8,339,856,101	8,282,311,094	27,700,621	46.92%	92.78%	21,177,603	76.45%

Raw reads, the total number of sequenced raw reads; Base Number, total base number in the clean data; Clean Reads, the number of pair-end Reads in the clean data; GC Content (%), the proportion of GC content in the clean data; Q30 (%), Q30 base percentage in the clean data; Mapped Reads, the number of mapped reads in the pair-end Reads; Mapped Ratio (%), the proportion of mapped reads in the clean reads

**Fig. 2:**
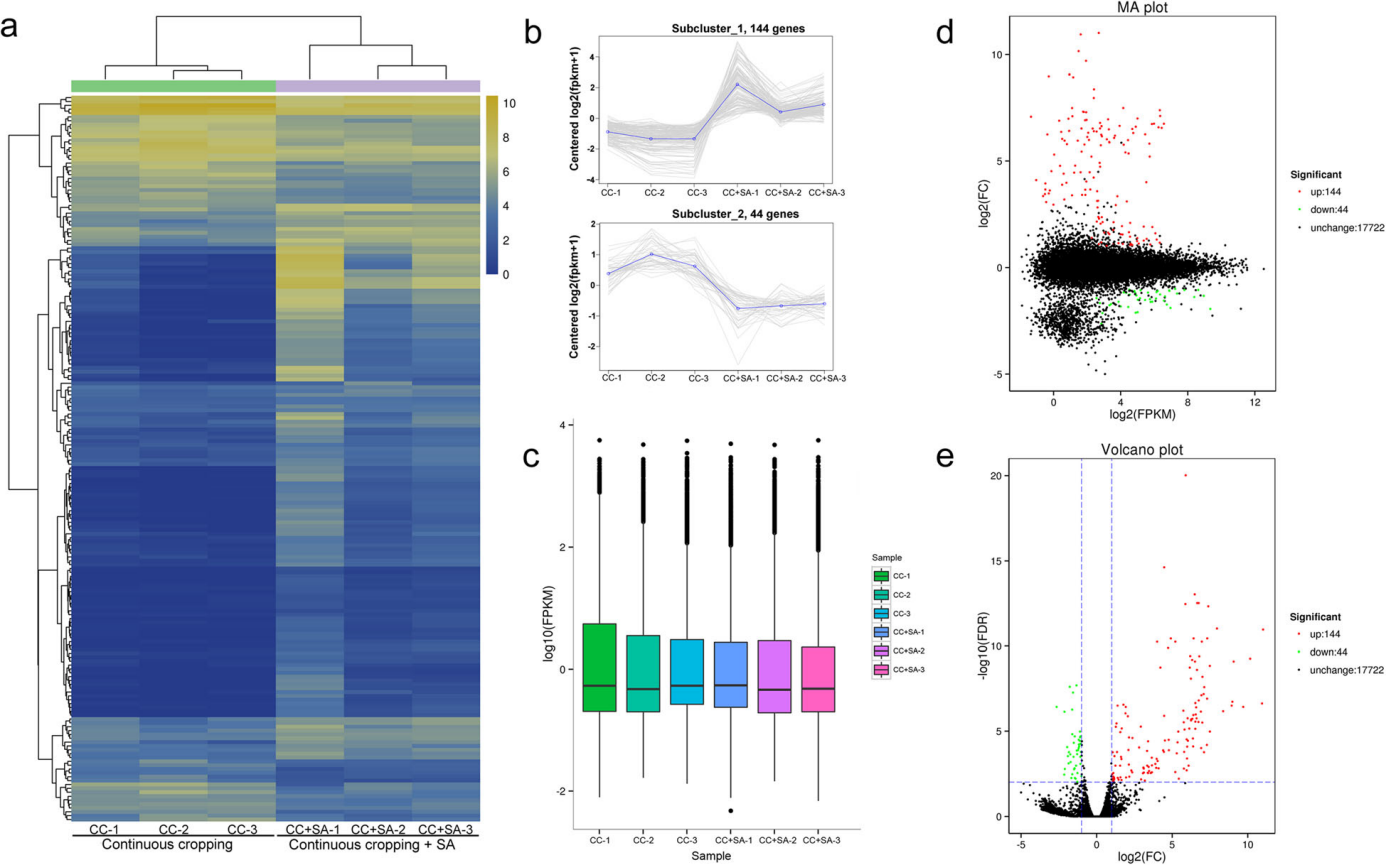
Expression analysis of the DEGs between CC and CC + SA roots. **a**-**b** Hierarchical cluster analysis. Different columns in the figure represent different samples, and different rows represent different genes. The colors from blue to yellow indicate gene expression from low to high, respectively. **c** Fragments per kilobase of transcript per million mapped reads (FPKM) boxplot. **d** MA plot. **e** Volcano plot. Each point in the MA plot and volcano plot represents a gene. The green points represent downregulated genes, the red points represent upregulated genes, and the black points represent unchanged genes

One of our primary goals was to identify the global changes in DEGs among different libraries. In this study, a total of 188 DEGs were identified between CC and CC + SA, including 144 upregulated and 44 downregulated genes (Fig. 2). The hierarchical cluster (H-cluster) analysis of all DEGs is shown in Fig. 2a-b. The results showed that all 188 DEGs were classified into 2 subclusters in both the SA treatment and control groups (Fig. 2b). The expression level in each sample was measured by the overall discrete level of expression (Fig. 2c). The overall distribution of the gene expression abundances and differential fold changes in the two groups are shown in an MA plot (Fig. 2d). The differences in gene expression level between the two groups and their statistical significances were visualized with a volcano plot (Fig. 2e).

### Functional classification of DEGs

**Fig. 3:**
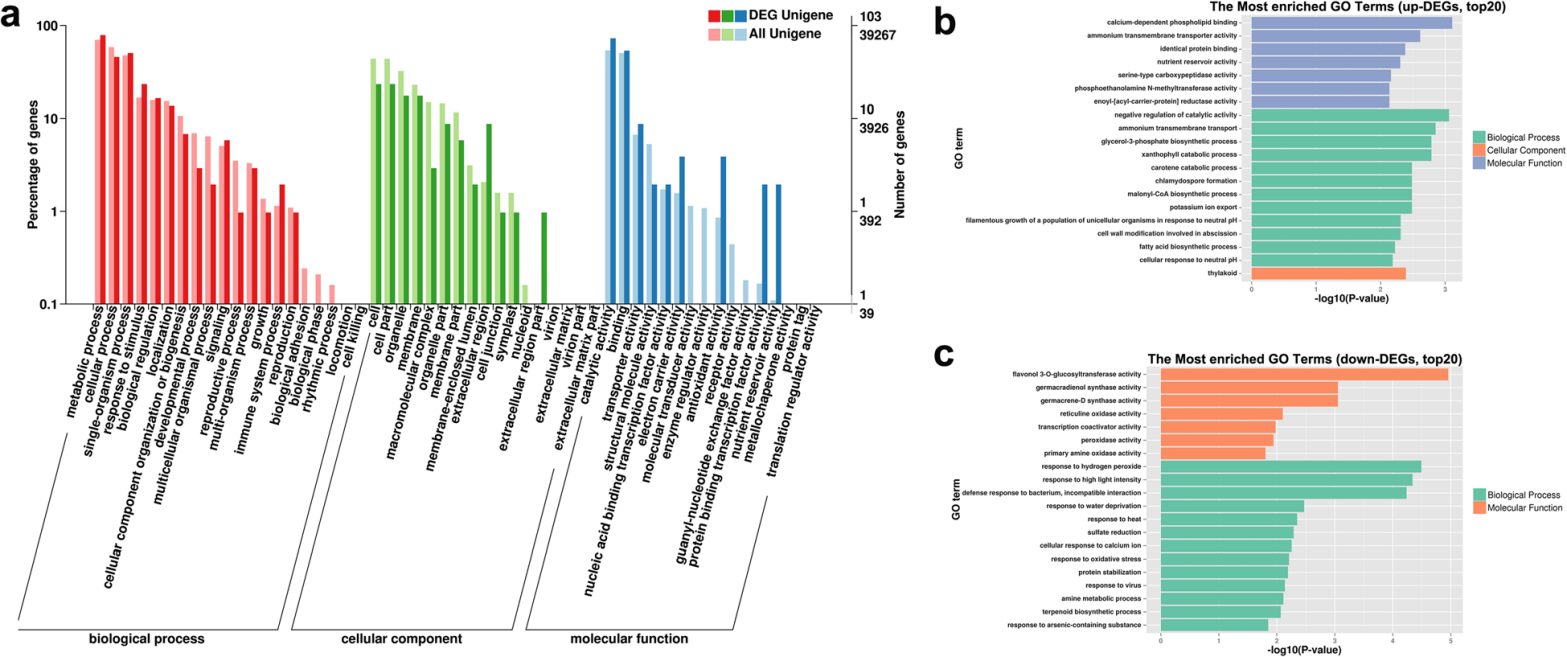
GO enrichment of DEGs. **a** GO enrichment of all DEGs. GO terms belonging to biological process, cellular component, and molecular function are shown in red, green and blue, respectively. **b**-**c** Significantly enriched GO terms (*P* < 0.05) among the up- and downregulated DEGs. GO terms belonging to biological process, cellular component, and molecular function are shown in green, orange and blue, respectively

To further investigate the functions of the DEGs, a GO term enrichment analysis of the DEGs was performed with GOseq. In this study, 103 identified DEGs were annotated with GO terms and assigned to the following three ontologies: biological process, cellular component, and molecular function. Most DEGs between CC and CC + SA were assigned to metabolic process, cellular process, single-organism process, response to stimulus, biological regulation and localization in the biological process group (red in Fig. 3a, Additional file 1: Table S2); cell, cell part, organelle, and membrane in the cellular component group (green in Fig. 3a, Additional file 1: Table S2); and catalytic activity and binding in the molecular function group (blue in Fig. 3a, Additional file 1: Table S2).

Specifically, the most highly enriched GO terms (top 20) among the up- and downregulated DEGs were analyzed with a corrected *p*-value < 0.05, as shown in Fig. 3b, c. Among the upregulated genes, many DEGs were assigned to nutrient transport–related GO terms, including ammonium transmembrane transport (GO:0072488) and potassium ion export (GO:0071435) in biological process and calcium-dependent phospholipid binding (GO:0005544), ammonium transmembrane transporter activity (GO:0008519), and nutrient reservoir activity (GO:0045735) in molecular function (Fig. 3b, Additional file 1: Table S3). The genes associated with nutrient transport–related GO terms are shown in Table 2, including two Ammonium transporter 3, two Annexin-like protein and two Germin-like protein genes (Table 2). Other upregulated DEGs were assigned to different GO terms, such as negative regulation of catalytic activity (GO:0043086), glycerol-3-phosphate biosynthetic process (GO:0046167), xanthophyll catabolic process (GO:0016124), and carotene catabolic process (GO:0016121) in biological process; identical protein binding (GO:0042802) and serine-type carboxypeptidase activity (GO:0004185) in molecular function; and thylakoid (GO:0009579) in cellular component (Fig. 3b, Additional file 1: Table S3). Interestingly, three of the GO terms, xanthophyll catabolic process (GO:0016124), carotene catabolic process (GO:0016121) and thylakoid (GO:0009579), play important roles in plant photosynthesis. Among the downregulated genes, many DEGs were associated with defense-related GO terms, such as response to hydrogen peroxide (GO:0042542), response to high light intensity (GO:0009644), defense response to bacterium, incompatible interaction (GO:0009816), response to water deprivation (GO:0009414), response to heat (GO:0009408), response to oxidative stress (GO:0006979), and response to virus (GO:0009615) in biological process and peroxidase activity in molecular function (Fig. 3c, Additional file 1: Table S4).

**Table 2:** The upregulated genes assigned to nutrient transport related GO terms

Gene ID	CC	CC + SA	FDR	log2FC	GO_annotation	nr_annotation
*c119123.graph_c0*	0.20 ± 0.12	19.44 ± 10.23	9.25E-14	6.49	GO:0072488; GO:0008519	Ammonium transporter 3 member 1-like
*c112306.graph_c0*	0.20 ± 0.17	14.54 ± 7.52	1.82E-09	6.16	GO:0072488; GO:0008519	Ammonium transporter 3 member 1-like
*c106508.graph_c0*	0.67 ± 0.34	8.46 ± 4.21	0.001	3.66	GO:0071435; GO:0005544	Annexin D1-like
*c116599.graph_c0*	0.3 ± 0.14	30.38 ± 18.53	3.03E-13	6.65	GO:0005544	Annexin-like protein RJ4-like isoform 2
*c120020.graph_c0*	3.77 ± 0.60	22.37 ± 7.96	5.42E-06	2.92	GO:0045735	Germin-like protein 2–1-like
*c107762.graph_c0*	0.25 ± 0.20	13.83 ± 7.51	6.26E-07	5.74	GO:0045735	Germin-like protein 9–3-like

**Fig. 4:**
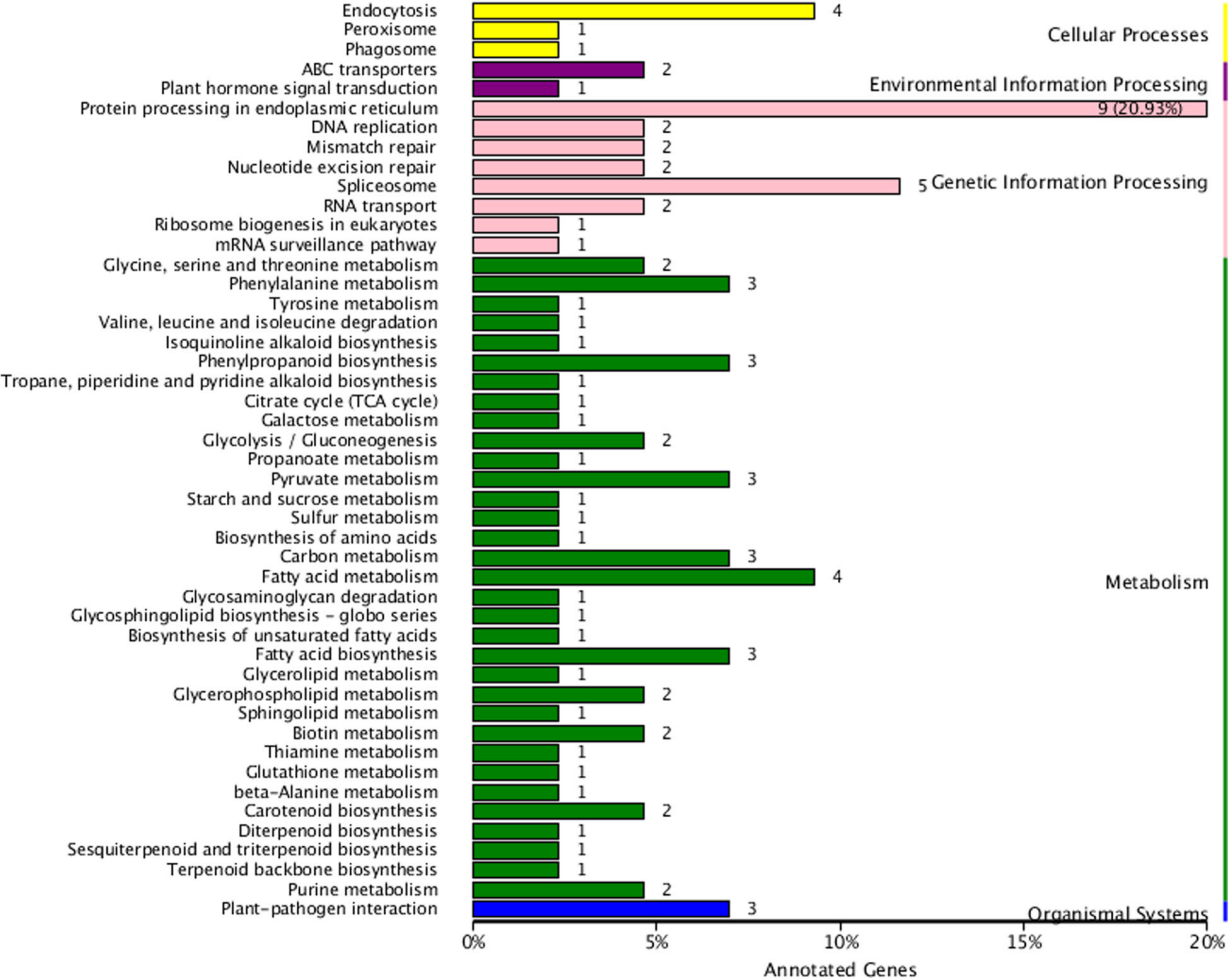
Significantly enriched KEGG pathways of DEGs. The number of DEGs annotated to a pathway is marked on the top of each bar

KEGG enrichment analysis was performed with KOBAS 2.0 to reveal both common and tissue-specific patterns of expression. The KEGG pathways of all the DEGs were classified into five main categories: cellular processes, environmental information processing, genetic information processing, metabolism and organismal systems. Among the most representative pathways, 9 DEGs were associated with protein processing in the endoplasmic reticulum (ko04141); 5 DEGs were assigned to spliceosome (ko03040) in genetic information processing; 4 DEGs were associated with endocytosis (ko04144) in cellular processes; 4 DEGs were assigned to fatty acid metabolism (ko01212) in metabolism; and 3 DEGs were assigned to plant-pathogen interaction (ko04626) in organismal systems (Fig. 4).

### Hsp-family genes associated with recovery strategies

**Fig. 5:**
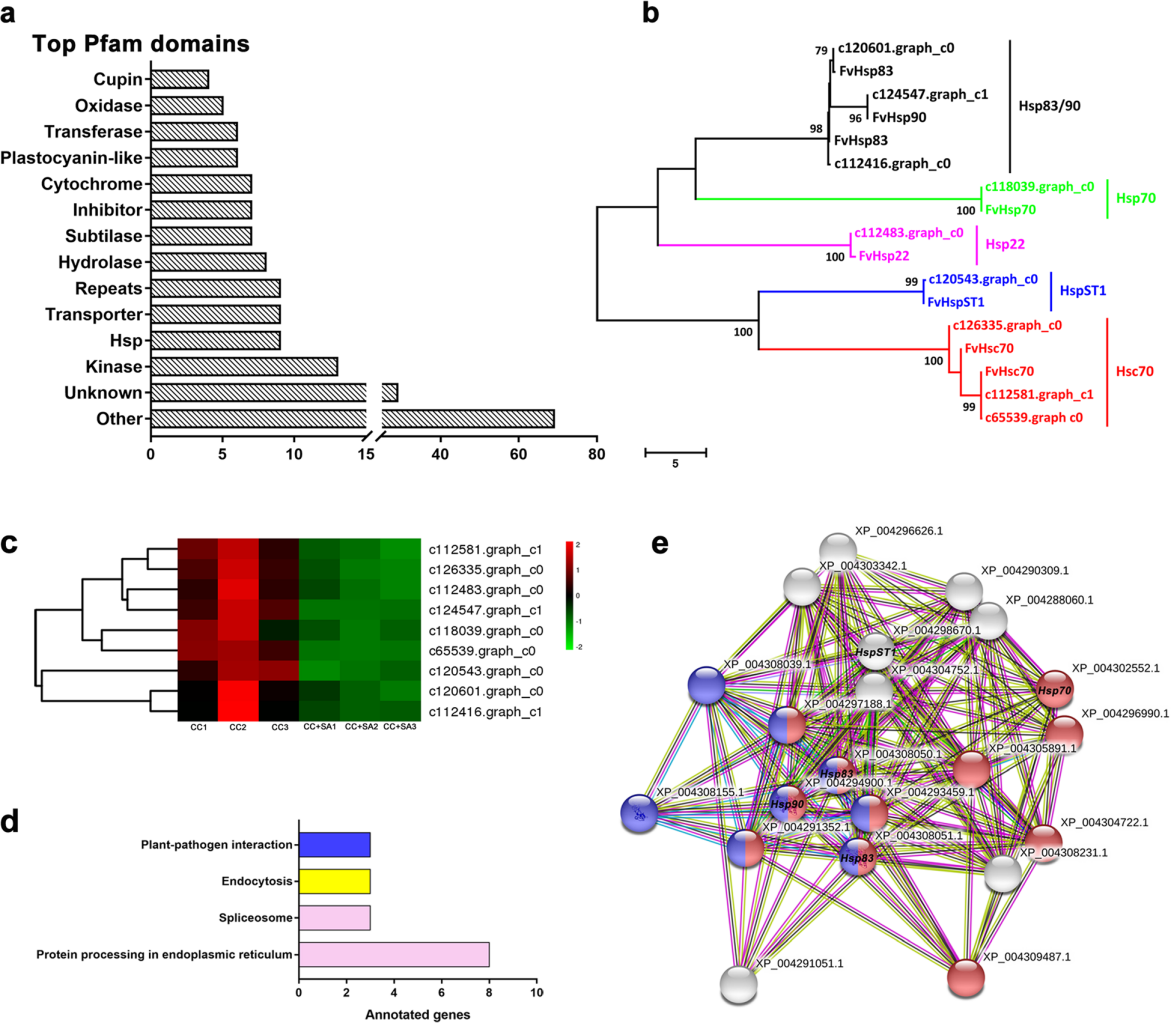
Comparative analysis of Hsp-family genes. **a** The top 14 Pfam domains in the DEGs. **b** A neighbor-joining gene tree of the Hsp-family genes found in the DEGs. The phylogenetic tree was constructed using MEGA 7.0 with 1000 bootstrap replications. **c** Heatmap of the Hsp-family genes differentially regulated in response to SA treatment. The colors from green to red indicate gene expression from low to high, respectively. **d** KEGG enrichment analysis of the Hsp-family genes found among the DEGs. **e** Interaction network of five Hsp-family genes regulated in response to SA treatment. Red indicates proteins involved in protein processing in the endoplasmic reticulum pathway, and blue indicates proteins involved in the plant-pathogen interaction pathway

We analyzed the Pfam protein domains of the DEGs to predict their functions (Fig. 5a). Kinase, Hsp, transporter, and repeat proteins were particularly abundant among the DEGs. Hsp-family proteins play important roles in plant tolerance mechanisms under different stresses. In our study, a total of 9 Hsps were found among all the DEGs. A gene tree was constructed for the 9 Hsp domain–containing proteins between *Fragaria × ananassa* Duch. and *Fragaria vesca*. We found that these nine *Hsp* genes belonged to *Hsp83* (*c120601.graph_c0*, *c112416.graph_c1*), *Hsp 90* (*c124547.graph_c1*), *Hsp70* (*c118039.graph_c0*), *HspST1* (*c120543.graph_c0*), and *Hsc70* (*c65539.graph_c0*, *c112581.graph_c1*, *c126335.graph_c0*) (Fig. 5b).

**Fig. 6:**
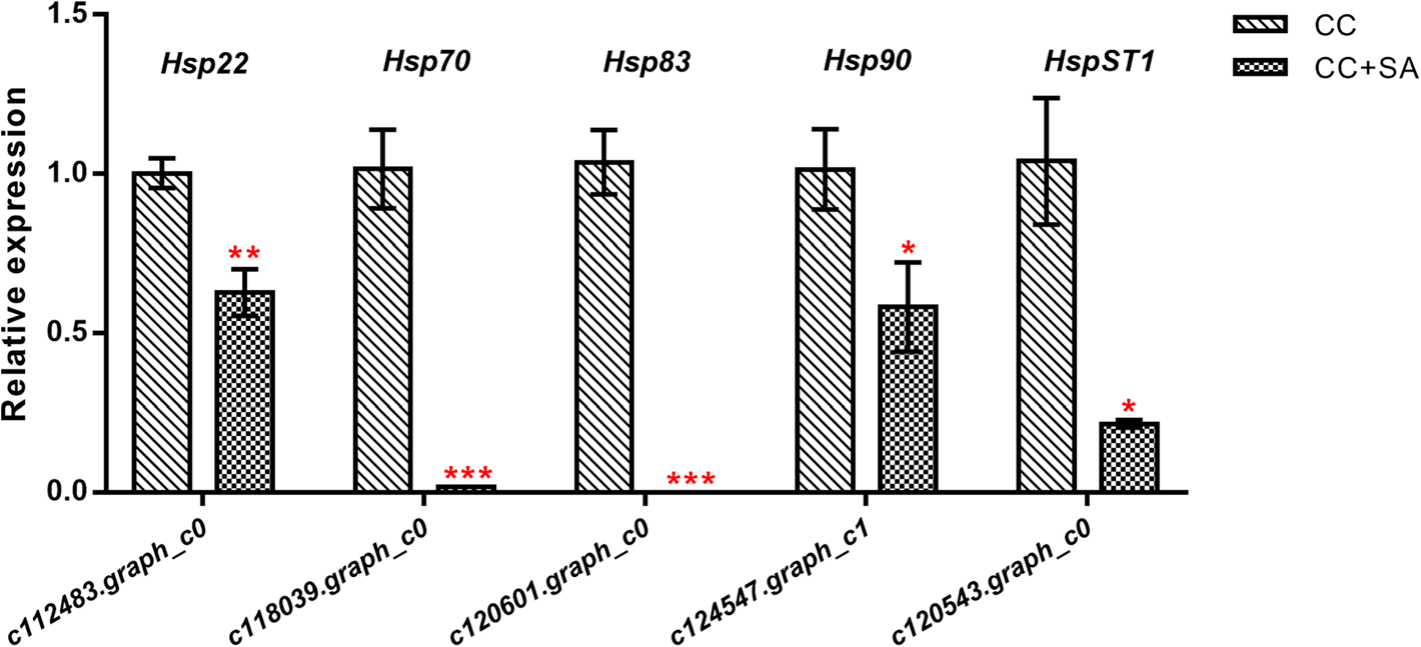
Expression profiles of five Hsp-family genes. The bars indicate the standard deviation. The data were normalized to a DNA-binding protein (EU727547) gene using the 2^*-*ΔΔCT^ method. * indicates *P* < 0.05, ** indicates *P* < 0.01, and *** indicates *P* < 0.001

We analyzed the expression patterns of these 9 *Hsp-*family genes to predict their functions in response to the SA. A heatmap of the 9 *Hsp-*family genes showed that they were all significantly downregulated after the SA was applied to the CC soil (Fig. 5c). The KEGG enrichment analysis of these 9 genes showed that all the genes except *c120543.graph_c0* (*HspST1*) were associated with protein processing in the endoplasmic reticulum (Fig. 6b, Additional file 1: Table S1). The three *Hsc70* genes were assigned to the spliceosome and endocytosis pathways, and the *Hsp83/90* genes were associated with plant-pathogen interactions (Fig. 5d, Additional file 1: Table S1).

The interaction network of the Hsps was analyzed to further understand their regulatory mechanisms. Five of these proteins (Hsp70, two Hsp83s, Hsp90, and HspST1) showed interactions with other proteins (Fig. 5e). Six proteins in the network were enriched in both the protein processing in the endoplasmic reticulum and plant-pathogen interaction pathways (represented by the circles with both red and blue in Fig. 5e).

### Real-time qPCR validation of gene expression profiles

To verify the RNA-Seq results, five Hsp-family DEGs were selected for qPCR detection, namely, *Hsp22* (*c112483.graph_c0*), *Hsp70* (*c118039.graph_c0*), *Hsp83* (*c120601.graph_c0*), *Hsp90* (*c124547.graph_c1*), and *HspST1* (*c120543.graph_c0*) (Fig. 6). The qRT-PCR results showed that all five of these *Hsp* genes were significantly downregulated in the CC + SA soil roots. Moreover, *Hsp70* and *HSP* could not be detected after the SA was applied to the CC soil roots. The qPCR data, in combination with the sequencing results, indicated that the RNA-Seq data were reliable.

## Discussion

CC obstacles are a major factor limiting strawberry production. Developed after years of hard work, our SA can effectively improve the growth of strawberry plants (Fig. 1). The SA contains many nutrients, such as sugar, amino acids and other high-energy compounds, which are important nutrient sources for crops and can be directly absorbed and utilized by strawberry plants [14]. Under continuous rotation, long-term monoculture of strawberry also results in the accumulation of autotoxic substances in the soil, thus inhibiting plant growth [3]. However, SA treatment could significantly reduce the accumulation of autotoxic substances in strawberry rhizosphere soil and alleviate the adverse effects of these autotoxic substances on strawberry plants [19]. Moreover, the SA might significantly increase the activities of urease, invertase, and polyphenol oxidase in the soil [14]. Overall, SA treatment could improve the CC soil environment and make it more conducive to plant growth.

Plants have evolved specific defensive strategies to face multiple environmental challenges [20]. It is still unknown what changes occur in strawberry plants in SA-treated CC soil, but it is imperative to understand the recovery mechanisms after application of the SA to the soil to improve CC tolerance through breeding. This paper provided genome-wide transcriptomic analyses of recovery responses in strawberry plants after the SA was applied to CC soil.

Comparative analyses of the RNA-Seq data from the SA-treated and control strawberry roots revealed a total of 188 genes that were differentially expressed (Fig. 2), including 144 upregulated and 44 downregulated genes. The most enriched GO terms among the up- and downregulated DEGs showed that many upregulated DEGs were associated with nutrient transport–related and photosynthesis-related GO terms (Fig. 3). In this study, six nutrient transport–related genes were identified, including ammonium transporter 3 members, Annexin-like proteins and Germin-like proteins (Table 2). Ammonium is a major source of nitrogen for plants, and ammonium transporters play vital roles in nitrogen metabolism [21, 22]. The annexin-like proteins are Ca^2+^-dependent phospholipid-binding genes in plants, and they might be involved in responses to various stress treatments [23]. Previous studies have shown that Germin-like proteins are involved in cell wall restructuring and osmotic regulation after different abiotic stress stimuli [24, 25]. Furthermore, some photosynthesis-related genes were also upregulated after SA treatment (Fig. 3b). Xanthophyll, carotene and thylakoids are necessary for photosynthesis in plants, and the upregulation of these genes is beneficial to plant growth. Meanwhile, many downregulated DEGs were assigned to defense-related GO terms (Fig. 3c). These changes in plant gene expression indicate a transition from defense to growth after SA treatment. This tradeoff between growth and defense is due to resource restrictions that demand prioritization of either growth or defense in plants [26]. When the SA was applied to improve the CC soil conditions, the plants began to reduce their defensive responses. The roots allowed the reallocation of resources to nutrient transport to support plant growth. This phenomenon can be further shown by the increase in strawberry plant biomass after SA treatment (Fig. 1).

KEGG is a database resource used for the systematic analysis of gene function [27]. KEGG pathway enrichment analysis of the DEGs revealed relationships among genomic, chemical and systemic functional information. In this study, 72 DEGs were mapped to KEGG pathways. The most highly represented pathways were endocytosis, protein processing in the endoplasmic reticulum, spliceosome, fatty acid metabolism, and plant-pathogen interaction (Fig. 4). Endocytosis plays important roles in many areas of cell and developmental biology and is a major pathway by which cells sense environmental changes [28]. Endocytosis is used by cells to internalize molecules and external nutrients, or it mediates the entry of invading pathogens into host cells [29, 30]. External materials can be internalized by the clathrin-dependent endocytosis pathway; subsequently, the external materials are engulfed in an endosome and delivered to the lysosome for degradation [28, 31, 32]. In addition, cells have evolved protein quality control systems to prevent or delay a myriad of diseases. Endoplasmic reticulum–associated protein degradation (ERAD) in the ER protein processing pathway is the most common way. ERAD ensures that misfolded proteins are detected and eliminated. The hypersensitive response (HR) is also among the most important plant defense reactions in plant-pathogen interactions [33, 34]. The HR was first reported for *Puccinia dispersa* by H. Marshall Ward in the twentieth century [35]; this process causes rapid cell death at the attempted invasion site, confines the invasive pathogens and sends signals to the plant that could activate additional defenses [34, 36].

**Fig. 7:**
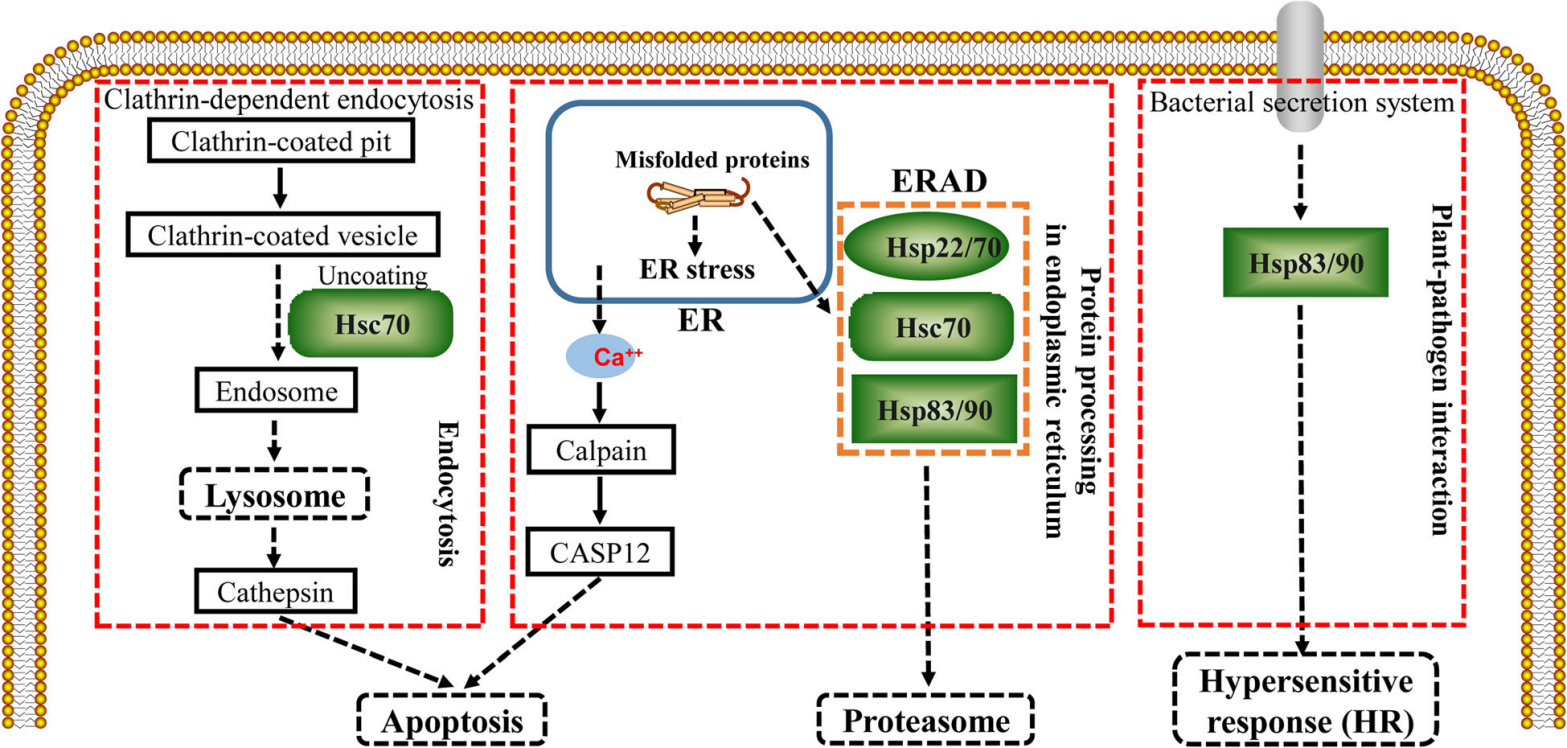
A possible functional network of Hsp-family genes in response to SA treatment. This figure was created through ScienceSlides 2005

Plants have evolved adaptive mechanisms to cope with multiple stresses. In this paper, the *Hsp* genes were found in several pathways highly enriched in DEGs, such as endocytosis, protein processing in the endoplasmic reticulum, spliceosome, and plant-pathogen interaction (Figs. 4, 5d and 7). All the Hsp-family DEGs, except for HspST1, are involved in protein processing in the endoplasmic reticulum (Fig. 7). Hsc70 participates in the process of uncoating clathrin-coated vesicles during their movement to the endosome (Fig. 7). *Hsp83/90*, which is involved in plant-pathogen interactions, might play a relevant role in the plant HR (Fig. 7). In plants, the *Hsp* genes not only are expressed under high-temperature stress but also play crucial roles in many developmental processes and in protecting plants against stresses [37–40]. Nathalie et al. showed that *HSP22* was induced by oxidative stress, indicating that downregulation of *FaHSP22* might be caused by a reduction in oxidative stress after SA treatment [41]. The Hsp70 family, including *Hsc70*, which is expressed constitutively, and *Hsp70*, which is induced by heat shock and stress, has essential functions in the appropriate folding and trafficking of nonnative proteins in the cell under both normal and stress conditions [40, 42]. *Hsp83* is active in *Nicotiana benthamiana* in response to *Sonchus yellow net virus* (SYNV) and *Impatiens necrotic spot virus* (INSV) [43]. *Hsp90* genes have been isolated from many plant species, and their major role is to manage protein folding, but they are also induced in adaptation to stresses, such as heat, cold, salt and heavy metal stress [40, 44–47]. These genes discussed above all play relevant roles in stress adaptation in plants.

Our previous study found that the CC obstacles in strawberry are attributable to an imbalance in plant nutrient availability and an accumulation of autotoxic substances, soil-borne pathogens and plant-parasitic nematodes, as well as other phenomena causing declines in soil health [2–5]. This study suggests that SA treatment might alleviate these problems and allow the CC soil ecology to recover, resulting in reduced stress on the plants. In strawberry roots, a reduction in external stresses leads to an overall decrease in defense-related gene expression, particularly that of the Hsp family. Then, the strawberry plants can reallocate resources to enact potential changes in the root, such as an increase in nutrient transport, to contribute to plant growth.

## Conclusion

The current study identified genes in strawberry roots that respond to SA treatment by differential expression analysis. Dramatic differences were found in the root transcriptomes of CC and CC + SA plants. The overall increase in nutrient transport–related genes and decrease in defense-related genes demonstrated that strawberry plants reallocated resources in favor of increased plant growth after SA treatment. The downregulation of 9 *Hsp* genes, which were mainly involved in plant defense pathways, such as protein processing in the endoplasmic reticulum, endocytosis, and plant-pathogen interaction, suggested additional recovery strategies in the SA-treated strawberry roots. The results in this paper contribute to a deeper understanding of the recovery strategies of strawberry roots after SA treatment in CC soil.

## Methods

### Plant materials and RNA extraction

The experiment used a typical cultivated strawberry variety, *Fragaria × ananassa* Duch. “Benihoppe”, was provided by Beijing Engineering Research Center for Strawberries and carried out at the Beijing Academy of Forestry and Pomology Sciences, Haidian District, Beijing, China (40°1´21″N, 116°16´32″E). The SA, with patent no. ZL200610112418.7, was mainly composed of insect body and insect metabolites and produced independently by our laboratory. All plants were cultivated at 22 ± 1 °C in greenhouses in a 16 h light/8 h dark photoperiod. The greenhouse contained 80 strawberry beds that were 100 cm in length× 40 cm in width× 40 cm in height; 40 strawberry beds were treated with the SA, and the other half served as a control. The strawberry seedlings were planted in two rows per bed. The greenhouses were subjected to general agricultural management practices, which included the following basic fertilizer applications: 29985 kg/ha farm manure with > 25% organic matter and 300 kg/ha NPK fertilizer with ≥45% N + P2O4 + K2O. The plant materials consisted of two groups: continuous cropping (CC) and continuous cropping with the soil amendment (CC + SA). The CC strawberry plants were planted in CC soil, which was monocultivated with strawberry plants for 12 years. The CC + SA strawberry plants were cultivated in the same CC soil in which the SA was applied once during strawberry engraftment. The plant samples were collected randomly 15 days after the SA treatment, and the roots were rinsed with water. The fresh weight was recorded immediately, and the root samples of the two groups (CC and CC + SA) were used for further RNA-Seq analysis. Each sample group included 3 biological replicates (CC-1, CC-2, and CC-3; CC + SA-1, CC + SA-2, and CC + SA-3), and the roots of 3 seedlings per treatment were pooled as one biological replication [48]. In total, 18 seedlings (3 seedlings × 3 biological replications × 2 treatments) were included in the analysis. All samples were snap-frozen in liquid nitrogen and stored at − 80 °C.

Total RNA was extracted from 100 mg of root tissue using a plant RNA Kit (BioTeke, Beijing, China) following the manufacturer’s specifications. First-strand cDNA was reverse transcribed using a FastQuant RT Kit (Tiangen, Beijing, China).

### RNA-Seq library construction and sequencing

A total of 1 μg of RNA per sample was used as input material for RNA sample preparation. Sequencing libraries were generated using a NEBNext UltraTM RNA Library Prep Kit for Illumina (NEB, USA). To preferentially select cDNA library fragments of 240 bp in length, the AMPure XP system (Beckman Coulter, Beverly, USA) was used to purify the fragments. Then, three microliters of USER Enzyme (NEB, USA) was mixed with the size-selected fragments. PCR was performed with Phusion High-Fidelity DNA polymerase, Universal PCR primers and Index (X) Primer. Finally, PCR products were purified (AMPure XP system), and library quality was assessed on the Agilent Bioanalyzer 2100 system. Clustering of the index-coded samples was performed on a cBot Cluster Generation System using a TruSeq PE Cluster Kit v4-cBot-HS (Illumina). After cluster generation, the library preparations were sequenced on an Illumina platform at Beijing BioMarker Corporation.

### Data analysis

The raw data (raw reads) in fastq format were processed through in-house Perl scripts. Adaptor sequences, reads containing poly-N and low-quality sequence reads were removed from the data sets. The raw sequences were transformed into clean reads after data processing. The Q30 and GC content of the clean data were calculated. The clean reads were then mapped to the reference genome sequence with TopHat2 tools software. A differential expression analysis of the two groups was performed using the DESeq R package (1.10.1). The *P*-values were adjusted using Benjamini and Hochberg’s approach to control the false discovery rate (FDR). Genes with an adjusted *P*-value < 0.01 and a |fold change (FC)| ≥ 2 according to DESeq were considered differentially expressed. Heatmaps and hierarchical clustering were generated with Genesis 1.8.1. A Gene Ontology (GO) enrichment analysis was implemented by the GOseq R package based on the Wallenius noncentral hypergeometric distribution [49]. KOBAS software was used to test the statistical enrichment of the DEGs in KEGG (http://www.genome.jp/kegg/ or http://www.kegg.jp/) pathways [27, 50]. The phylogenetic trees constructed by MEGA 7.0 using the neighbor-joining (NJ) method with 1000-fold bootstrap resampling [51, 52]. SMART (http://smart.embl.de/) was used to derive the interaction network [53]. The data were analyzed by an independent-samples T test using SPSS 20.0 (SPSS Inc., USA).

### Gene expression analysis

The *Hsp*-family genes were selected for real-time qPCR validation using a QuantStudio™ 6 Flex Real-Time PCR System (Applied Biosystems, Foster City, CA, USA). The qRT-PCR primers were obtained from qPrimerDB (https://biodb.swu.edu.cn/qprimerdb/) and are listed in Additional file 1: Table S1 [54]. Each reaction mixture included 10 μl of 2 × SYBR® Select Master Mix (Applied Biosystems, Foster City, CA, USA), 100 ng of diluted cDNA product, and 0.8 μl of each of the two primers (10 μM), and was adjusted to 20 μl with DNase/RNase-free water. Gene expression was presented as relative units after standardization to the strawberry housekeeping gene DNA-binding protein (DBP) EU727547 as an internal control using the 2^-ΔΔCT^ method with three technological replicates [55–57]. Each reaction was repeated using three independent biological and technical replicates.

## Supplementary information

**Additional file 1:** **Table S1.** Primers used for q-PCR, **Table S2.** GO enrichment of all DEGs, **Table S3.** Most enriched GO Terms among the upregulated DEGs. **Table S4.** Most enriched GO Terms among the upregulated DEGs.

## Data Availability

The raw transcriptomic data generated during the current study are available from the corresponding author on reasonable request. All other data generated or analyzed during this study are included in this published article and its supplementary information files.

## Abbreviations

CC: Continuous cropping
cDNA: Complementary DNA
DEGs: Differentially expressed genes
FC: Fold change
FDR: False discovery rate
GO: Gene Ontology
Hsc: Heat shock cognate protein
Hsp: Heat shock protein
KEGG: Kyoto Encyclopedia of Genes and Genomes
SA: Soil amendment
